# Targeting essential pathways in trypanosomatids gives insights into protozoan mechanisms of cell death

**DOI:** 10.1186/1756-3305-3-107

**Published:** 2010-11-17

**Authors:** Despina Smirlis, Michael Duszenko, Antonio Jiménez Ruiz, Effie Scoulica, Patrick Bastien, Nicolas Fasel, Ketty Soteriadou

**Affiliations:** 1 Laboratory of Molecular Parasitology, Department of Microbiology, Hellenic Pasteur Institute, 127 Bas. Sofias Ave., 11521 Athens, Greece; 2 Interfaculty Institute for Biochemistry (IFIB), University of Tübingen, Tübingen, Germany; 3 Departamento de Bioquímica y Biología Molecular, Campus Universitario, Universidad de Alcalá, 28871 Alcalá de Henares, Madrid, Spain; 4 Department of Clinical Bacteriology, Parasitology, Zoonoses and Geographical Medicine, Faculty of Medicine, University of Crete, Heraklion, Greece; 5 University Montpellier 1, UFR Médecine, Laboratoire de Parasitologie-Mycologie, 163 rue Auguste Broussonet, F-34090 Montpellier, France; 6 Department of Biochemistry, 155 Chemin des Boveresses, University of Lausanne, Epalinges CH-1066, Switzerland

## Abstract

Apoptosis is a normal component of the development and health of multicellular organisms. However, apoptosis is now considered a prerogative of unicellular organisms, including the trypanosomatids of the genera *Trypanosoma spp*. and *Leishmania spp*., causative agents of some of the most important neglected human diseases. Trypanosomatids show typical hallmarks of apoptosis, although they lack some of the key molecules contributing to this process in metazoans, like caspase genes, Bcl-2 family genes and the TNF-related family of receptors. Despite the lack of these molecules, trypanosomatids appear to have the basic machinery to commit suicide. The components of the apoptotic execution machinery of these parasites are slowly coming into light, by targeting essential processes and pathways with different apoptogenic agents and inhibitors. This review will be confined to the events known to drive trypanosomatid parasites to apoptosis.

## Introduction

From the mid-nineteenth century, many observations have indicated that cell death plays a considerable role during physiological processes of multicellular organisms, particularly during embryogenesis and metamorphosis [1]. The term programmed cell death (PCD) was introduced in 1964, proposing that cell death during development is not of accidental nature but follows a sequence of controlled steps leading to locally and temporally defined self-destruction [2], in contrast to necrosis, which is a form of cell-death that results from acute tissue injury and provokes an inflammatory response. It is evident that death may occur through different mechanisms leading to distinct morphologies.

Consequently, different types of PCD have been described, the most important forms being apoptosis and autophagic cell death [3]. The term apoptosis describes biochemical processes and morphological features leading to controlled cellular self-destruction such as rounding-up of the cell, condensation of the chromatin, fragmentation of the nucleus (karyorhexis), loss of the mitochondrial membrane potential (ΔΨm), plasma membrane blebbing, and others [4], whereas autophagy is the type of cell death that occurs without chromatin condensation, but often accompanied by massive autophagic vacuolization of the cytoplasm [5]. In mammalian cells the two major apoptotic pathways are the ''intrinsic'' pathway, involving mitochondrial membrane permeabilisation regulated by the members of the Bcl2/Bax protein family, and the transmembrane ''extrinsic'' pathway comprising of activation of death receptors (DRs), via the TNF superfamily of DRs [6]. Despite the fact that these two pathways are relatively distinct, their co-existence and cross-talk is also possible [7]

Although it was initially assumed that apoptosis arose with multicellularity, there is now increasing experimental evidence that similar mechanisms are operative in trypanosomatids of the genera *Trypanosoma spp. (T. brucei *and *T. cruzi) *and *Leishmania spp*. These parasites display complex life cycles, with multiple differentiation forms alternating between mammalian and insect hosts. Trypanosomatids are the causative agents of diseases such as Kala-azar (visceral leishmaniasis), cutaneous and mucocutaneous leishmaniasis, Chagas disease (American trypanosomiasis) and African sleeping sickness (African trypanosomiasis), diseases affecting more than 27 million people worldwide [8].

Different types of cell death exist in these unicellular parasites, including apoptosis and autophagic cell death (reviewed in [9,10]), triggered in response to diverse stimuli. In trypanosomatids, the former is induced by different stimuli such as heat shock [11-14], reactive oxygen species (ROS) [15-23], antiparasitic drugs [10,24-65], prostaglandins [66], starvation [67-69], antimicrobial peptides[70,71], antibodies [72], serum as a source of complement [19,73], and mutations in cell-cycle regulated genes [74] (See additional file 1: Table S1). Once apoptosis is triggered, a cascade of events common to mammalian apoptosis takes place such as production of reactive oxygen species (ROS) and lipid peroxidation, increase in cytosolic Ca^2+ ^levels, changes in mitochondrial membrane potential (ΔΨm), exposition of phosphatidylserine in the outer leaflet of the plasma membrane, maintenance of an intact plasma membrane until late stages of the process, release of cytochrome c, and induction of proteases and DNA cleavage (reviewed in [75,76]) (See additional file 1: Table S1).

Although these trypanosomatids show the common outcomes of apoptosis as compared with mammalian apoptosis, the absence of homologues to mammalian key regulatory or effector molecules of apoptosis (like TNF-related family of receptors, Bcl-2 family members and caspases) indicates that the pathways of apoptosis are in part distinct in these divergent eukaryotes. However, despite the lack of these molecules, trypanosomatids appear to have the basic machinery to commit suicide.

Trypanosomatids also possess a functional autophagic system (reviewed in [77-79]) that appears to be essential for differentiation and for parasite maintenance and survival [67,80,81], being activated during differentiation, starvation-induced stress [67,80-82], treatment with different drugs [10,83-85] and antimicrobial peptides [86-88]. Although autophagy may also lead to cell death, it is generally regarded as a catabolic survival mechanism.

For this reason, this review will not describe autophagic cell death in trypanosomatids but will be confined to the components of the basic machinery that these parasites possess to commit suicide, and the pathways and/or biological processes that, when deregulated, drive these protozoan parasites to die in a controlled manner. Obeying the recommendations of the nomenclature commission of cell death [3], we use the term apoptosis here for an induced cell death in trypanosomatids that shows a considerable number of apoptosis hallmarks.

## Mitochondrial dysfunction in trypanosomatid apoptosis

Mitochondria have a central role in metazoan apoptotic cell death, as they are involved in the active control of apoptosis at several levels including the release of proapoptotic proteins [89]. The dysfunction of mitochondria is one of the hallmarks of apoptosis often associated with changes in ΔΨm-a key indicator of mitochondrial function that maybe either a consequence of or an early requirement for apoptosis [90,91].

**Figure 1:**
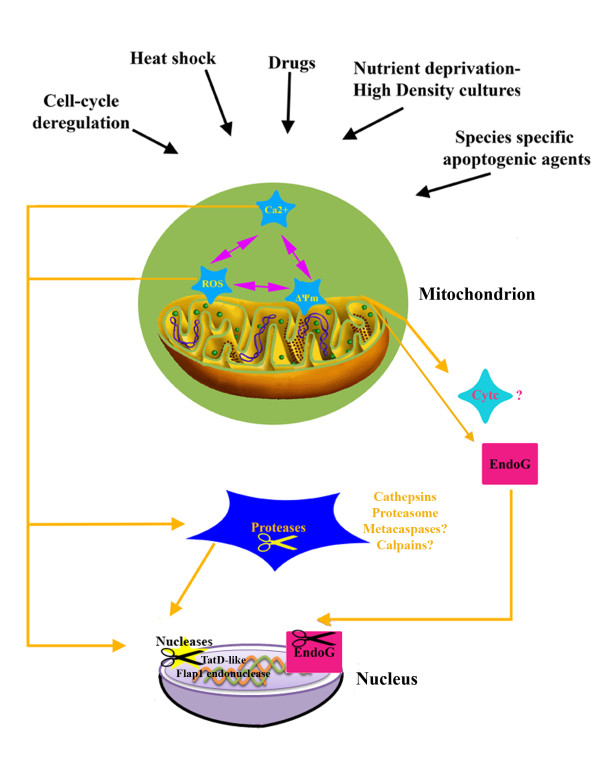
**Representation of the major pathways leading to apoptosis in trypanosomatids**. The different triggers of apoptosis result in the loss of mitochondrial membrane potential (ΔΨm), the generation of reactive oxygen species formation (ROS) and increase in cytosolic Ca^2+ ^(Ca^2+^). These changes potentiate the release of cytochome c and EndoG into the cytoplasm and the activation of proteases and nucleases to dismantle the parasites in an ordered fashion. Upon release from the mitochondrion EndoG translocates to the nucleus to degrade DNA. The question marks (?) represent a function that either awaits confirmation in trypanosomatids or that has been suggested for only some genera of trypanosomatids.

In trypanosomatids too, many apoptogenic agents or stresses, are associated with a dysfunction of the unique mitochondrion of these organisms indicated by the changes in ΔΨm (See additional file 1: Table S1). In this respect, the antiparasitic activity of many drugs is mediated by the loss of mitochondrial ΔΨm (See additional file 1: Table S1). Not only drug treatment but also physiological stress conditions may lead to apoptosis with changes in ΔΨm, for example: nutrient deprivation in stationary phase *L. donovani *promastigote cultures [92], heat stress in *L. infantum *promastigotes [12], high density cultures producing prostaglandin D2 in *T. brucei *[17,66], or prolonged endoplasmic reticulum (ER) stress in *T. brucei *parasites [93] (Figure 1, See additional file 1: Table S1).

The precise and coordinated function of the trypanosomatid mitochondrial respiratory chain complexes is a prerequisite for sustaining the proper mitochondrial potential (reviewed in [94]). Hence, inhibitors of respiratory complexes II [95,96] and III [96] resulted in the dissipation of ΔΨm, whereas complex I inhibition in *L. donovani *promastigotes, resulted in mitochondrial membrane hyperpolarisation [96]. On the contrary, increase in respiration rates and the concomitant generation of superoxide radicals by heat shock also induced apoptosis in *L. infantum *promastigotes and resulted in mitochondrial membrane hyperpolarisation [12]. Interestingly, this indicates that both an increase and a decrease in respiration and both hyperpolarisation and loss of ΔΨm maybe linked with apoptosis in trypansomatids demonstrating the importance of maintenance of proper ΔΨm in these parasites [96] (Table 1). Although the precise mitochondrial contribution to apoptosis awaits further elucidation the above data reinforce the conception that mitochondrial dysfunction and apoptosis in trypanosomatids are closely associated.

**Table 1:** Proteins in essential for survival processes, mitochondrial changes and effectors associated with trypansomatid apoptosis

Proteins in essential processes associated with apoptosis	Mitochondrial changes associated with apoptosis	Effectors of apoptosis
**Proteins associated with the cell-cycle** **Prohibitin **(*T. brucei*) **RACK **(*T. brucei*) **QM **(*T. brucei*) **PKC **(*L. donovani*) **GSK-3 **(*L. donovani*) **CRK3 **(*L. donovani*)	**Respiration and ΔΨm** Increase, Decrease	**Proteases** **Cathepsin-like proteases **(CPA, CPB, CPC) (*L. major, L. mexicana*)
**Proteins associated with proliferation and cell division** **Centrin **(*L. donovani*) **EF-1α **(*T. cruzi*)	**Mitochondrial membrane permeability** **ROS **by lipid peroxidation^b^ **ΔΨm **by mitochondrial matrix condensation^a^ **Bcl-2 **functional homologues^b^	**Proteasomal proteases** (*L. amazonensis*)
**Proteins involved in DNA replication** **Topoisomerases **(*Leishmania spp*.)	**Mitochondrial release of pro-apoptotic proteins** **Cytochrome c**^c^ **EndoG**	**Metacaspases** (*T. cruzi*, *L. donovani*)
**Proteins involved in nucleocytoplasmic transport** **Ran **(*T. brucei)* **RanBP1 **(*T. brucei)* **CAS **(*T. brucei)* **NTF-2 ***(T. brucei)*	**ΔΨm, ROS and Ca2+ interplay** Trigger → Cytosolic Ca2+ elevation → Ca2+ entry into mitochondrion → Dissipation of ΔΨm → ROS	**Nucleases** **EndoG** (*Leishmania spp*.)
**Proteins in cytoskeletal dynamics** **Tubulins^a^** (*L. donovani, T. brucei*)	Trigger → ROS → lipid peroxidation-→ elevation of cytosolic Ca2^+ ^→ Dissipation of ΔΨm	**TatD-like **endonuclease (*L. donovani*)
**Proteins in other processes** **SIR2 deacetylase** *(L. infantum*)	Trigger → ROS → ΔΨm → intracellular Ca2+ elevation	**Flap-endonuclease-1** (*L. donovani*)

a. Apoptosis is triggered by agents interfering with tubulin polymerization

b. Awaits confirmation

c. Unknown function in trypanosomatid apoptosis

In brackets, the genus or the species involved.

Mitochondrial dysfunction and apoptosis are often initiated in mammalian cells by the mitochondrial outer membrane permeabilisation (MMP) regulated by proapoptotic Bcl-2 family members [97]. No Bcl-2 family members with significant similarity to their mammalian counterparts are encoded by the genome of trypanosomatids, although functional homologues of these proteins may exist in trypanosomatids. To address this question, Arnoult *et al*. mixed human recombinant Bax- a Bcl-2 family member that induces MMP in mammalian cells via interaction with antiapoptotic family members [98,99] - with *L. major *intact mitochondria [100]. Bax was able to induce pore formation in intact *L. major *mitochondria, followed by a rapid release of cytochrome c [100]. In addition, ectopic expression of mammalian members of the Bcl-2 family, i.e. the antiapoptotic Bcl-X_L _and the proapoptotic Hrk, either reduced or increased the number of *L. infantum *promastigotes undergoing apoptosis respectively, upon treatment with eldefosine [24]. Bcl-X_L _was also able to partially reverse heat-shock induced cell death in *L. infantum *promastigotes [11]. In line with the above, ectopic expression of Bax in *T. brucei *induced loss of ΔΨm and release of cytochrome c [101]. Evidence therefore suggests that functional homologues of Bcl-2 family members may exist in trypanosomatids (Table 1). Overall, it appears that apoptosis maybe initiated from the mitochondrion in trypanosomatids, by certain death signals, as it occurs in metazoan mitochondrial mediated apoptosis.

## Calcium, ΔΨm, and ROS interplay in trypanosomatid apoptosis: a mitochondrial love-hate triangle

ROS-products formed during the normal metabolism or resulting from xenobiotic exposure- and Ca^2+ ^signals together are involved in a variety of vital cell functions and thus are necessary for cell survival. However, more recently it has become clear that cellular ROS and Ca^2+ ^overload can cause cytotoxicity and trigger either apoptotic or necrotic cell death [102,103]. ROS species and imbalance of calcium homeostasis, also contribute to apoptosis in trypanosomatids, and this occurs via more than one pathway (Figure 1, Table 1; reviewed in [76,94]).

In the first pathway, the trigger induces cytosolic Ca^2+ ^elevation. Ca^2+ ^enters the mitochondrion and dissipates ΔΨm. Preceding or following the ΔΨm, ROS are generated by mitochondria via oxidative phosphorylation. These events finally lead to the execution of apoptosis. This pathway has been reported in *T. cruzi *epimastigotes when exposed to fresh human serum [19]. Upon exposure to fresh human serum, the assembly membrane attack complex resulting from complement activation, allowed the influx of Ca^2+^, and consequently excessive mitochondrial Ca^2+ ^accumulation [19]. Mitochondrial Ca^2+ ^overload resulted in the loss of ΔΨm and increased superoxide anion production [19]. This connection between mitochondrial Ca^2+ ^overload and ROS generation has also been demonstrated in mammalian systems [104-106].

A second pathway has been described in which the trigger induces ROS formation, which in turn results in lipid peroxidation. Lipid peroxidation affects membrane fluidity and/or the function of calcium channels, disturbing the calcium homeostasis and thus resulting in the elevation of cytosolic Ca^2+ ^and the loss of ΔΨm. This in turn induces the execution of apoptosis. This pathway has been unraveled by treating *L. donovani *promastigotes with thenoyltrifluoro-acetone (a respiratory chain complex II inhibitor) [96], camptothecin or curcumin [26,51] or with hydrogen peroxide [20]. ROS production resulted in an elevation of the cytosolic Ca^2+ ^levels due to opening of non-selective and L-type voltage-gated ion channels in the plasma membrane [20,26,51]. Elevation of cytosolic calcium levels led to the uncoupling of mitochondrial oxidative phosphorylation, to the release of cytochrome c into the cytosol and directed promastigotes to follow the executionary pathway of apoptosis [15,26,51]. In *T. brucei*, ROS production has been reported to generate Ca^2+ ^homeostasis imbalance. However ROS production in this parasite impaired the mitochondrial Ca^2+ ^transport and disrupted the Ca^2+ ^barrier between nuclear envelope and cytosol. Consequently, in response to the extracellular stimulus, the mitochondrion did not accumulate Ca^2+ ^efficiently, leading to accumulation of excess Ca^2+ ^within the nucleus and induction of apoptosis thereafter [22].

Another model of mitochondria-induced apoptosis predicts the rapid loss of the mitochondrial potential by ROS, whereby calcium homeostasis is disturbed after dissipation of ΔΨm. This occurred with intracellular *L. donovani *amastigotes treated with potassium antimony tartrate. Potassium antimony tartrate generated ROS that was primarily concentrated in the macrophage parasitophorous vacuoles. ROS induced the loss of ΔΨm, which finally led to an elevation of Ca^2+ ^concentrations in both parasite and host cells [57].

In most cases, it seems that there is a correlation between ROS formation, calcium imbalance and loss of ΔΨm (Figure 1, Table 1). However, in other instances loss of ΔΨm led to apoptosis without the involvement of ROS or the change in Ca^+2 ^concentration. For example, neither ROS nor Ca^2+ ^ions were involved in dissipation of ΔΨm and apoptosis in *L. donovani *promastigotes treated with Aloe Vera extracts [28].

To protect themselves against oxidative stress, trypanosomatids possess both non-enzymatic (e.g. glutathione, trypanothione [107], ovothiol A [108]) and enzymatic scavengers. Unlike mammalian cells, trypanosomatids do not encode for the two key antioxidant enzymes catalase and glutathione peroxidase [109]. However, hydrogen peroxide metabolism is based on the trypanothione peroxidase system working in concert with NADPH and trypanothione; and scavenging of ROS from this system is required for parasite survival and infectivity [110-112]. Superoxide ions are detoxified by superoxide dismutase [113] and ROS from the mitochondrial respiratory chain by ascorbate peroxidase [114]. *L. major *promastigotes over-expressing ascorbate peroxidase showed enhanced tolerance to apoptosis mediated by oxidative stress [114]. Pteridine reductase (PTR1) has a function in essential pteridine salvage as well as in antifolate resistance [115]. The leishmanial PTR1 has been shown to protect intracellular amastigotes against reactive oxygen and nitrogen intermediates' toxicity, while PTR1-/- null mutants survived less well in macrophages [115]. Furthermore the targeting of this enzyme by a glycosyl dihydropyridine analogue induced apoptosis in *L. donovani *promastigotes [31] and intracellular amastigotes [116]. Likewise, expression of trypanosome alternative oxidase (TAO) [117] was increased under low-temperature stress; and inhibition of TAO was associated with apoptosis in the bloodstream form [118]. Protection of TAO against cell death was associated with protection from ROS generated by drugs like antrycide in TAO over-expressing transgenic *T. brucei *[23].

Non-metabolic enzymes have also been shown to protect parasites against oxidative stress. The surface lipophosphoglycan (LPG) of *Leishmania *[119] was suggested to scavenge oxygen radicals and LPG-deficient mutants were indeed more sensitive to ROS [120]. Moreover the cellular chaperone HSP70 has also been proposed to have a protective role against ROS. HSP70 appeared to be upregulated in *Leishmania *parasites undergoing heat shock, or in parasites treated with a sublethal dose of menadione, generating superoxide and hydrogen peroxide respectively [121] or with a nitric oxide donor [122]. Transfection of promastigotes with HSP70 caused a heat-inducible increase in resistance to peroxide [121]. The authors suggest that HSP70 upregulation is a mechanism for resisting toxic oxidants [121].

## Downstream of mitochondrial changes: the execution of apoptosis

The events that follow the disruption of ΔΨm result in protease and nuclease activation, responsible for dismantling the respective cells. The main executors of apoptosis in mammalian cells are a group of cysteine proteases, the caspases [123]. However, in the past few years, accumulating evidence in the literature supports the existence of pathways of caspase-independent apoptosis with central players proteases being cathepsins, calpains, granzymes A and B and the proteases of the proteasome [124]. Trypanosomatids do not have caspase genes, and therefore they undergo a caspase-independent apoptosis.

An example of a non-caspase executor of apoptosis involves the proteasomal proteases in *L. amazonensis *amastigotes (Table 1) treated with *NO donors [125]. Other putative executors of apoptosis are metacaspases (MCAs) (Table 1), i.e. cysteine proteases with similar folds as caspases [126]. The genome of *T. brucei *possesses five metacaspases (*Tb*MCA1-5) [127], whereas two genes are present in *T. cruzi *(*Tc*MCA3, *Tc*MCA5) [128], two in *L. donovani *(*Ld*MC1 and *Ld*MC2) [129] and one *in L. major *(*Lmj*MCA) [130,131]. These proteases have arginine/lysine proteolytic activity, and are unable to cleave caspase-specific substrates [129,131,132]. Heterologous expression of *Tb*MCA4 in yeast caused loss of respiration competence and clonal cell death [127], whereas the *L. major *metacaspase could replace the endogenous yeast metacaspase YCA1 in apoptosis [131]. The role of metacaspases as executors of apoptosis in trypanosomatids still remains controversial. For example, it has been suggested that the two *T. cruzi *MCAs might be involved in human serum-induced apoptosis [128], and that over-expression of *L. donovani *MCAs renders the parasites more sensitive to hydrogen-peroxide [129]. While *Tb*MCAs (2, 3 and 5) and the *L. major *metacaspase could play a functional role in key steps of the cell-cycle and division [130,133], their function in trypanosomatid apopotosis awaits confirmation.

Although caspases are not present in the trypanosomatid genomes, many investigators have reported the presence of caspase-like activity assessed by the cleavage of caspase-specific substrates and the inhibitory effect of caspase-specific inhibitory peptides [15,26,44,46,49-51,68,92,114,134,135]. This activity was described in *Leishmania *parasites treated with different drugs [92], hydrogen peroxide [15], inhibitors of protein kinase C [49,100], and in *T. cruzi *epimastigotes treated with human serum [135], as well as stationary phase or nutrient deprived parasites [68]. Therefore it is evident that proteases with little homology, but with overlapping activity to metazoan caspases, may be involved in the execution of apoptosis in trypanosomatids. Indeed, Zangger *et al*. showed that cleavage of the caspase-specific substrate, a DEVD peptide in a 10 day axenic culture, was inhibited by E-64, an inhibitor of cathepsin-like cysteine proteases [69] that does not inhibit caspases [126]. Moreover the DEVDase activity was not present in a double mutant of the cathepsin L-like cysteine CPA/CPB proteases [136], indicating that this activity is likely due to one of the two cysteine proteases [69]. In addition, the intracellular binding of the cell permeate pancaspase inhibitor Z-VAD-FMK, upon heat shock induced apoptosis, was attributed to the binding to the cathepsin B-like cysteine proteinase c (CPC) [137]. CPC was not only shown to bind z-VAD but also its knocking out appeared to make parasites survive better when exposed hydrogen peroxide [137], therefore providing strong evidence that at least part of the execution of apoptosis in *Leishmania spp*. may function via the involvement of CPC [137]. Finally cruzipain, the major cysteine protease of *T. cruzi*, was able to act on caspase substrates at low rates [138]. Overall these data suggest that the caspase substrate activity in trypanosomatids may be stimulated by the lysosomal cathepsin-like proteases (Table 1).

Using protease inhibitors, several investigators have demonstrated that proteases stimulate nucleases to degrade DNA. This was shown with the cysteine protease inhibitor E-64 in staurosporine treated *L. donovani *promastigotes [100] and with caspase inhibitors in *Leishmania *and *Trypanosoma *upon different triggers of cell death [15,44,46,51,135]. However, there are many examples of apoptosis, where DNA fragmentation was shown to be insensitive to caspase inhibitors or to caspase-like activity [22,28,32,47,54,66,69,139,140], suggesting that DNA degradation may be under the control of multiple proteases.

Although DNA fragmentation is commonly observed in trypanosomatids undergoing apoptosis, effectors of this pathway have only recently been described (Table 1). From the genome data it is known that trypanosomatids do not contain homologues of caspase-activated DNAase (CAD), one of the best characterised nucleases in mammalian apoptosis. In addition to CAD, mammalian cells possess a mitochondrial endonuclease G (EndoG) (Table 1) that translocates to the nucleus during caspase-independent apoptosis [141,142]. EndoG is encoded in the trypanosomatid genome [140,143,144], as a mitochondrial enzyme [140,144] that upon oxidative [114,143] and/or drug induced apoptosis [32,140,144], translocates to the nucleus (Figure 1; [143,144]). This enzyme, in the nucleus, forms separate complexes with Flap endonuclease-1 and TatD-like nuclease to generate the degradosome in *L. donovani *promastigotes [140]. Over-expression of this endonuclease strongly promoted apoptotic cell death under oxidant or differentiation-induced stress in *Leishmania*, while conversely down-regulation of EndoG conferred resistance to oxidative induced cell death in *T. brucei *[143], indicating that it is an essential effector of apoptosis in trypanosomatids.

During activation of apoptosis, ions and pH may play an important role in the execution process, affecting both nuclease and protease activity. This was demonstrated in camptothecin-treated *L. donovani *promastigotes, where treatment of the drug was followed by a significant decrease in intracellular pH and the impairment of the Na^+^-K^+ ^ATPase pump by oxidative stress [51]. The reduction of the K^+ ^concentration and the pH change propagated the protease activity (DEVDase) of untreated cytosolic *L. donovani *extracts [51]. Moreover, a nuclease present within the nuclei of untreated extracts of *L. donovani *that became activated in the presence of Mg^2+ ^and/or Ca^2+ ^ions was strongly repressed at physiological concentrations of K^+ ^[51]. The authors suggested that K^+ ^efflux from the cells during apoptosis is an important regulator of the nuclease activity [51]. Different ion requirements were observed for the nuclease activity from stationary phase *L. major *parasites that was inhibited by Zn^2+ ^ions, and was not dependent on Ca^+2 ^or Mg^2+ ^ions, although the addition of Mg^2+ ^ions improved this activity [69]. These differences in ion concentrations suggest that more than one nuclease is present in *Leishmania spp*. that is induced by different apoptosis triggers. In addition the *L. infantum *EndoG required Mg^2+^, Mn^2+ ^or Co^2+ ^ions for optimal activity, whereas moderate K^+ ^concentrations (150 mM) or higher Na^+ ^concentrations (300 mM) inhibited the enzyme [144]. Therefore, imbalances of intracellular ion concentrations and pH values during apoptosis in trypanosomatids may trigger the caspase-independent activation of proteases and nucleases to execute cell death.

## Apoptosis is associated with deregulation of essential biological processes and protein functions in trypanosomatids

The induction of apoptosis in mammalian cells is often associated with alterations of essential biological processes. For example the tight coupling of proliferation and cell-cycle control with apoptosis, provides a means by which an organism can regulate cell expansion and is imperative for cellular homeostasis. Hence the deregulation of the cell-cycle may result in apoptosis in mammalian cells (reviewed in [145,146]). The relationship between cell-cycle control and apoptosis is now becoming evident in trypanosomatids (Table 1). A hint to such a relationship came from experiments, where *T. brucei *parasites were treated with the lectin concanavalin A (ConA). ConA used the major cell-surface glycoprotein as a ligand, and induced both cell-cycle defects [147] and apoptosis [148]. In addition, in apoptotic parasites treated with ConA, there was a differential expression of genes whose homologues are known to be involved in cell-cycle control in mammalian cells, like prohibitin, the trypanosome receptor for activated protein C (RACK) [149,150]and the homologue of the QM protein (a regulator of the c-jun protooncoprotein) [149,151].

Kinases also provide an important link between cell-cycle coordination and apoptosis. This was demonstrated by the inhibition of glycogen synthase-3 short isoform (*Ld*GSK-3s) and CRK3 (the CDK1 homologue in *Leishmania spp*.) (Table 1) [65]. The indirubins, 6-Br-indirubin-3'-oxime and 6-Br-5-methylindirubin-3'oxime, that show selectivity against CRK3 and *Ld*GSK-3 s respectively, induced apoptosis in *L. donovani *promastigotes [65]. In mammalian cells, CDK1 was shown to be an essential component of certain forms of apoptosis (reviewed in [146]) and provided the functional link between mitotic arrest and apoptosis [152]. In addition, mammalian GSK-3 had a pro-apoptotic action for the intrinsic signalling pathway by the facilitation of signals that cause disruption of mitochondria [153]. Therefore the above observations suggest that the events downstream of GSK-3 and CDK1 and their respective homologues, might be different in mammalian cells and in trypanosomatids. On the contrary staurosporine (prototypical ATP-competitive kinase inhibitor) and withaferin A (potent protein kinase C inhibitor), known apoptogenic agents for mammalian cells [154-156], also induce apoptosis in *L. donovani *parasites [49,100] implying that inhibition of homologous kinases may induce the execution of apoptosis in both trypanosomatids and mammals.

Evidence exists, that proteins associated with proliferation and cell division may be linked to apoptosis in trypanosomatids, as in higher eukaryotes (Table 1). The knocking down of centrin in *L. donovani *amastigotes, encoding a cytoskeletal calcium binding protein that regulates cytokinesis in trypanosomatids [74,157], induces apoptotic death [74]. In addition, elongation-factor 1 α, (EF-1α) a protein involved in eukaryotic protein biosynthesis and proliferation [158,159], translocates from the cytoplasm to the nucleus, in apoptotic *T. cruzi *epimastigotes [160]. The authors suggest that the nuclear translocation may confer a distinct function to this protein and that *Tc*EF-1α could participate in the regulation of expression of genes involved in the control of cell death in *T. cruzi *[160]. Nevertheless, for assessing an active role of *Tc*EF-1α in apoptosis, as occurs with the mammalian homologue [161,162] further investigations are required.

Among the genes that have been implicated in the protection against apoptosis are the Silence Information Regulator 2 (Sir2) genes [163]. Homologues of the proteins are classified as NAD dependent deacetylases [164]. Sir2 proteins are hypothesized to play a key role in an organism's response to stresses (such as heat or starvation) and to be responsible for the life-extending effects of calorie restriction mediated by decreased cAMP and thus lowered protein kinase A signalling [164]. *L. infantum *amastigotes over-expressing the Sir2 homologue, showed a striking increase in the survival rate due to an inherent resistance to apoptosis [165]. Furthermore, sirtinol, a commercially available inhibitor of SIR2 deacetylases, significantly inhibited the *in vitro *proliferation of *L. infantum *axenic amastigotes in a dose-dependent manner and induced apoptosis [166]. Promastigotes that over-expressed the gene also showed an increase in viability under starvation conditions [165]. Taking into account the above observations, it is tempting to speculate that *Leishmania *SIR2 can participate among other factors in the control of cell death (Table 1), and can interact with cellular factors necessary for the cell death machinery [167].

Other genes essential for parasite viability whose inhibition leads to apoptosis in trypanosomatids are topoisomerases (Table 1). Topoisomerases are enzymes that use DNA strand scission, manipulation and rejoining activities to deal with DNA torsional stress, which makes them potential targets for treating parasitic diseases. As topoisomearases are involved in replication, transcription, chromosomal condensation and segregation, inhibitors of these enzymes are expected to interfere with these functions and to have a drastic inhibitory effect on the growth of trypanosomatid parasites [168-172]. The topoisomerase inhibitors berberine [47], camptothecin [51,52], dihydrobetulinic acid [173], baicalein [140], Hoechst 33342 [174], novobiocin [56], pentamidine, doxorubicin [55] luteolin, and diospyrin [38] induce apoptosis, thus providing support that there is a direct correlation between topoisomerase inhibition and apoptosis. The best studied example of apoptosis described in the previous sections has been obtained with camptothecin [51,52], a well characterised topoisomearse IB inhibitor (reviewed in [175]).

The trypanosomatid microtubule cytoskeleton has also been shown to be associated with apoptosis when deregulated. In this respect, agents that interfere with microtubule dynamics (Table 1) including taxol [176] and certain alkaloids [45] induce apoptosis in trypanosomatids. In mammalian cells, the link between microtubules, microtubule interfering agents and apoptosis is mainly associated with modifications of biological processes (M phase arrest) and signalling pathways (mitotic spindle assembly checkpoint activation, Bcl-2 phosphorylation, c-Jun NH2-terminal kinase activation) which ultimately lead to the accumulation of signals required for the engagement to cell death (reviewed in [177]). As trypanosomatids lack many of the signalling and effector molecules that regulate apoptosis and key cell-cycle checkpoints (reviewed in [178]), the pathways leading to apoptosis-like death upon exposure to microtubule interfering agents are most likely to be different. A possible mechanism for the action of these drugs may be associated with disruption of microtubule networks within the mitochondrion [177] or via the direct opening of the permeability transition pore [179].

Apart from deregulation of the cytoskeleton, apoptosis is induced by the inhibition of active nuclear transport [180] (Table 1). Ran-GTPase, a small GTPase that was first discovered to be essential in nucleocytoplasmic transport, is now known to regulate a variety of processes such as mitotic spindle assembly, nuclear envelope assembly, cell-cycle progression and the mitotic checkpoint in mammalian cells [181-184]. In *T. brucei*, RNAi-mediated gene silencing of Ran and of several of its partners RanBP1, CAS, and NTF2 -the latter having a function solely in nucleocytoplasmic transport- induced apoptosis [180], therefore indicating that impairment of this transport is an intrinsic signal for triggering apoptosis in trypanosomatids [180]. This was later confirmed in mammalian cells by Wong *et al*. who found that active disruption of nuclear trafficking was an important part for promoting apoptosis before the wholesale breakdown of the nuclear envelope and mixing of the cytosolic and nuclear compartments [185].

## The Spliced Leader RNA silencing pathway: a novel player in endoplasmic reticulum stress induced apoptosis

Accumulation of unfolded proteins in the lumen of the endoplasmic reticulum (ER) results in changes of Ca^2+ ^homeostasis, inhibition of glycosylation, oxidative stress and exposure to reducing agents [186]. This induces a coordinated adaptive program called the unfolded protein response (UPR). The UPR alleviates stress by upregulating protein folding and ER associated protein degradation (ERAD) and by inhibiting protein synthesis [186]. However, when protein misfolding is persistent or excessive, ER stress triggers cell death, typically apoptosis [187]. Several mechanisms have been proposed for linking the distressed ER to cell death in Metazoa including direct activation of proteases, kinases, transcription factors, and Bcl-2 family modulators [186,187].

**Figure 2:**
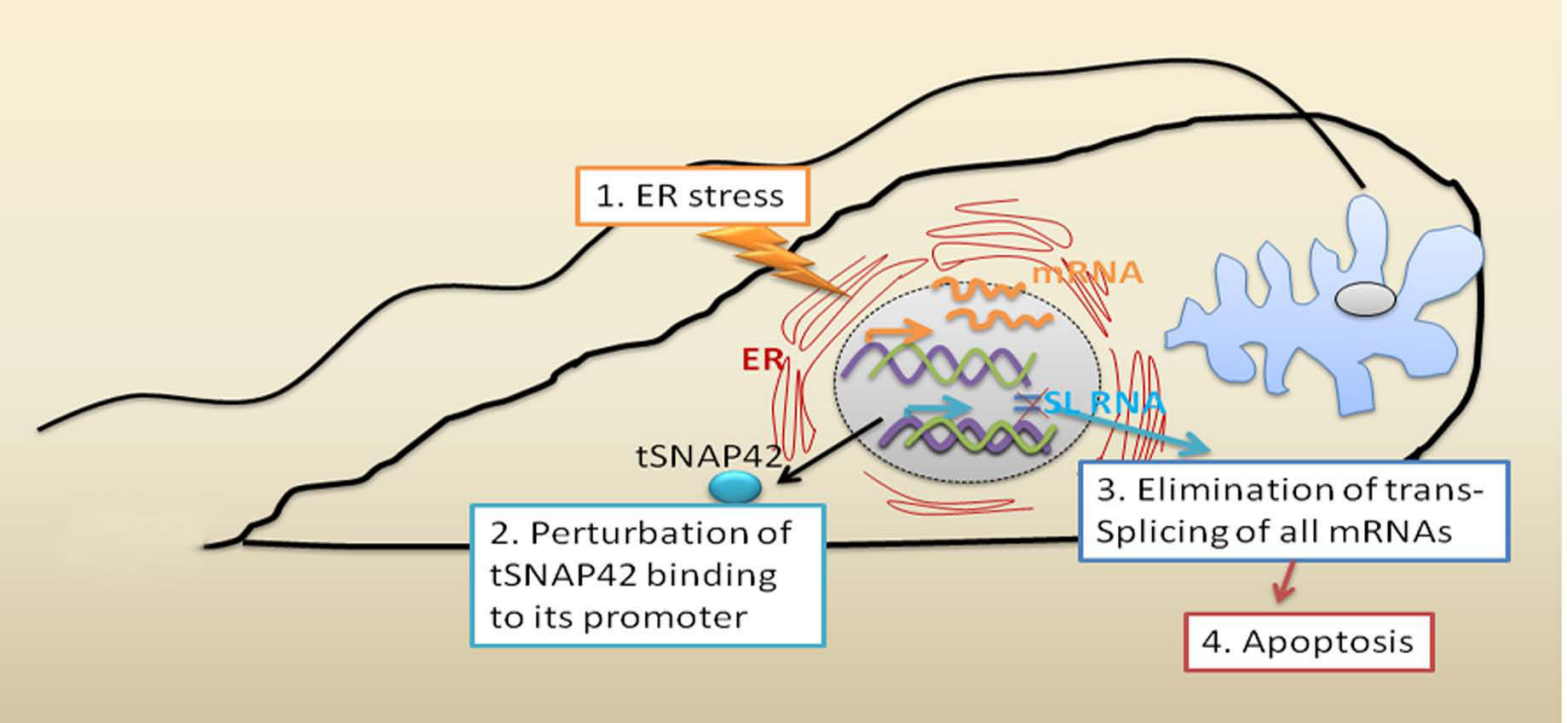
**Representation of the ER stress induced Spliced Leader RNA Silencing pathway leading apoptosis in *T. brucei***. Trypanosomatid parasites share a gene expression mode which differs greatly from that of their human and insect hosts. In these unicellular eukaryotes, protein coding genes are transcribed polycistronically and individual mRNAs are processed from precursors by spliced leader (SL) trans-splicing and polyadenylation. In trans-splicing, the SL RNA is consumed through a transfer of its 5'-terminal part to the 5'-end of mRNAs. Since all mRNAs are trans-spliced, the parasites depend on strong and continuous SL RNA synthesis mediated by RNA polymerase II and transcription factors like tSNAP42. Upon prolonged ER stress (1. ER stress), the binding of tSNAP42 to its cognate promoter, the promoter element of the Spliced Leader RNA (SL RNA) is perturbed (2. Perturbation of tSNAP42 binding to its promoter). This leads to the shutting off of SL RNA transcription and the elimination of trans-splicing of all mRNAs (3. Elimination of trans-splicing of all mRNAs). The SL RNA silencing pathway finally induces apoptosis (4. Apoptosis).

Trypanosomes lack factors that induce UPR, however upon ER stress, transcriptome changes occur in the procyclic form of *T. brucei*, primarily via differential mRNA stabilisation, that are similar to those induced by conventional UPR in metazoans and yeast [93]. The ER stress response triggered by the presence of the reducing agent dithiothreitol (DTT), induced the shutting off of Spliced Leader (SL) RNA transcription by perturbing the binding of the transcription factor tSNAP42 to the SL RNA promoter (Figure 2; [188]), leading to Spliced Leader RNA silencing (SLS). The SLS pathway was also induced by other stresses such as those derived from differences in pH or silencing of relevant proteins such as the signal-recognition particle receptor [188], SEC63 (a protein participating in protein translocation across the ER membrane) or SEC61 (the translocation channel) [93]. The SLS triggered a form of cell death in the parasite, reminiscent of apoptosis (Figure 2), having outcomes like, exposition of phosphatidylserine in the outer leaflet of the plasma membrane, cytoplasmic [Ca^2+^] elevation, reduction in ΔΨm and ROS formation, as well as ATG8-YFP puncta, indicating the induction of autophagy [93]. The authors proposed that the SLS serves as a unique death pathway, replacing caspase-mediated apoptosis observed in higher eukaryotes [93]. This mechanism of cell death has only been demonstrated in *T. brucei*, but homologues of the transcription factors that regulate SL transcription are also present in the other trypanosomatids [189] thus making it a possible trypanosomatid-specific apoptosis pathway.

## Conclusions

Trypanosomatids appear to possess an endogenous basic machinery that drives the cells to die in a regulated manner. These unicellular organisms encode homologues of metazoan proteins that control the cell-cycle and cell differentiation, including proto-oncogenes, cyclin and cyclin-dependent kinases, that when deregulated result in mitotic catastrophes and apoptosis [190]. However, trypanosomatids lack the classical metazoan effectors of apoptosis (the typical death receptors, caspases, Bcl-2 family members and p53). Although the nature of the pathways that result in the execution of apoptosis may not exactly recapitulate that of mammalian systems, they represent a simple and valuable model which will assist in the future understanding of the complex connections between apoptotic and non-apoptotic mammalian cell death pathways.

In addition to a better understanding of the evolution of the molecular mechanisms of apoptosis, the physiological relevance of this process in these unicellular organisms has attracted much attention. A possible role of apoptosis in the biology of trypanosomatids would be to control parasite numbers in response to limited resources, or within the host for the perpetuation of the infection [191]. It could also be a useful mechanism to avoid an inflammatory response leading to killing of the entire parasite population [69,92]. Apoptosis of *Leishmania spp*. allows the silencing in human PMNs enabling the intracellular survival of non-apoptotic parasites [192]. In addition, the regulation of apoptosis could allow a stringent coupling of appropriate cell differentiation with cell survival [193]. Furthermore, another possible role of apoptosis in these parasites would be the maintenance of clonality and assurance of propagation only of the cells fit to transmit the disease [194].

Finally our ability to unravel the pathways important for apoptosis in these protozoa and to predict the consequences of altering specific components of the larger network will provide us with tools to develop novel treatments for combating the devastating diseases caused by these parasites.

## List of abbreviations

ATG: AuTophaGy; Bad: BclX_L_/BCL2 associated death promoter homolog: Bax: Bcl-2-associated × protein; Bcl-2: B-cell lymphoma 2; Bcl-XL: BCL2L protein: long form of Bcl-x; Bid: BH3 interacting domain death agonist; CAS: CAS: cellular apoptosis susceptibility; CPA: cysteine peptidase A; CPB: cysteine peptidase B; CPC: cysteine peptidase C; CRK3: Cdc2p related protein kinase 3; DR: death receptor; EF-1α: elongation factor 1 alpha; EndoG: endonuclease G; Hrk: activator of apoptosis harakiri; HSP: heat shock protein; ROS: reactive oxygen species; MMP: permeabilisaton of mitochondrial membranes; NGF-IB: Nerve growth factor-IB; *NO: nitric oxide; NTF-2: nuclear factor 2; PMN: human polymorphonuclear leucocytes; RACK: receptor for activated C-kinase; Ran: RAs-related Nuclear protein; RanBP1: Ran binding protein 1; RNAi: RNA interference; TNF: Tumour Necrosis Factor; UPR: unfolded protein response; YFP: yellow fluorescent protein; Z-VAD-FMK: carbobenzoxy-valyl-alanyl-aspartyl-[O-methyl]-fluoromethylketone; ΔΨm: mitochondrial membrane potential.

## Competing interests

The authors declare that they have no competing interests.

## Authors' contributions

DS participated in the conception, design, acquisition and interpretation of data and coordination as well as to draft the manuscript. KS participated in the design, conception, acquisition and interpretation of data, and helped to draft the manuscript. MD, AJR, ES, PB NF have participated in the acquisition and interpretation of data, and have participated in drafting and revising the manuscript. All authors read and approved the final manuscript.

## Supplementary Material

Additional file 1 **Table S1 -Inducers, cellular responses and markers of apoptosis in trypansomatids**. The table lists known triggers/inducers of apoptosis in trypanosomatid species, as well as the parasites' cellular responses to the above triggers, and markers of apoptosis. Click here for file
