# Supplementary material for: Targeting essential pathways in trypanosomatids gives insights into protozoan mechanisms of cell death
Source: Parasit Vectors. 2010 Nov 17;3:107. doi: 10.1186/1756-3305-3-107 (PMC3136144; doi:10.1186/1756-3305-3-107)
Supplement: Additional file 1 — Table S1 -Inducers, cellular responses and markers of apoptosis in trypansomatids. The table lists known triggers/inducers of apoptosis in trypanosomatid species, as well as the parasites' cellular responses to the above triggers, and markers of apoptosis. [file 1756-3305-3-107-S1.DOC]

**Additional file 1**

|  | **Inducer/Trigger** | **Cellular responses and markers of apoptosis** |
| --- | --- | --- |
| ***L. infantum*** | **Drugs:**  Eldefosine [24]  Miltefosine [63, 64]  Sirtinol [166] in axenic amastigotes  Trans-platinum complexes [41]  **Other triggers:**  Heat shock [11, 12]  Polyamine derivatives in promastigotes [59] | Cell shrinkage [64]  PS externalisation [11, 41, 59, 64]  Maintenance of plasma membrane integrity [11, 41]  Decrease in ΔΨm [24, 59, 64]  Increase in ΔΨm [12, 24]  Release of cytochrome c[64]  Activation of cellular proteases[64]  Caspase-like activity [11]  DNA fragmentation [24, 59, 64, 166]  DNA ladder formation [11, 63]  Chromatin condensation [63]  Elevation of superoxide anion [24]  Dyskinetoplasty [64] |
| ***L. donovani*** | **Drugs/Natural products:**  Aloe Vera extracts [28]  Amphotericin B [92]  Artemisin [53]  Baicalein [140]  Berberine chloride [47]  Camptothecin [51, 52]  Curcumin [26]  Depsipeptide IB-01212 [71]  Dihydrobetulinic acid [173]  Diindolylmethane [46]  Diospyrin [38]  Doxorubicin [55]  Geldanamycin during amastigote to promastigote differentiation [33]  Glycosyl dihydropyridine analogue [31, 116]  Hoechst 33342 [174]  Indirubins: 5-Me-6-BIO and 6-BIO [65] Luteolin [37]  Miltefosine [44]  Nelfinavir [32]  Novobiocin [55]  Pentamidine [55]  Pentostam in axenic amastigotes [92]  Potassium antimony tartrate in intracellular amastigotes [57]  Quercetin [37]  Racemoside A [29]  Sodium m-arsenite [30]  Taxol [176]  Withaferin A [49]  **Other triggers:**  Centrin deletion in axenic amastigotes [74]  Heat shock [14]  Hydrogen peroxide [15, 20]  Respiratory chain inhibition [71, 96]  Stationary phase culture [92] | Cell shrinkage [15, 29, 44]  PS externalisation [26, 28, 31, 37, 38, 47, 55-57, 65, 96]  Maintenance of plasma membrane integrity [30, 51, 71, 95, 173]  Decrease in ΔΨm [28, 29, 31, 32, 46, 49, 51, 52, 55, 57, 71, 95, 116, 140]  Decline in intracellular ATP [46, 51, 71, 95]  Release of cytochrome c [26, 28, 49, 51, 55, 56]  Activation of cellular proteases [53, 55]  Caspase-like activity [15, 46, 49, 56, 74, 92]  PARP-like cleavage [15, 51]  EndoG release from mitochondria [32, 140]  DNA fragmentation [26, 28-33, 38, 44, 46, 47, 57, 65, 71, 92, 95, 116, 173, 174, 176]  DNA ladder formation [15, 28, 30, 38, 44, 46, 47, 49, 56, 92, 173, 176]  Chromatin condensation [15, 28, 29, 56, 65, 71, 92]  Elevation of ROS [26, 32, 33, 46, 47, 49, 51, 57, 96, 140]  Elevation of intracellular Ca^2+^ [20, 26, 51, 57, 96, 140]  Decrease in glutathione levels [20, 33]  Decrease in thiol equivalents [47]  Cell-cycle deregulation [26, 31, 33, 37, 53, 65, 74] |
| ***L. major*** | **Drugs:**  Staurosporine [100]  **Other triggers:**  Antimicrobial peptides [70]  Stationary phase culture [69] | Cell shrinkage [100]  PS externalisation [70]  Maintenance of plasma membrane integrity[100]  Decrease in ΔΨm [70, 100]  Decline in ATP production [70]  Caspase-like activity [70, 100]  Activation of cellular proteases [70, 100]  Cytochrome c release [100]  DNA fragmentation [69, 70, 100]  DNA ladder formation [69, 100]  Chromatin condensation [100]  Elevation of intracellular Ca^2+^ [70] |
| ***L. amazonensis*** | **Natural plant products:**  Yangambin [39]  **Other triggers:**  Heat shock [13]  Nitric oxide [125] | Morphological changes [13, 39]  Activation of proteasomal proteases [125]  DNA fragmentation [13, 125]  Elevation of intracellular Ca^2+^ [13]  Chromatin condensation [39]  Cell-cycle deregulation [39]  Formation of concentric membranous structures inside the mitochondria [39]  Cytoplasm acidification [39] |
| ***T.brucei*** | **Drugs:**  Alkaloids [45]  Quercetin [34]  **Other triggers:**  Hydrogen peroxide [22]  Inhibition of active nuclear transport by RNAi gene silencing of RanGTPase and its partners [180]  Lectins [147-150]  Persistent ER stress [93]  Prostandlandin D2 and metabolites of the J series [17, 66] | Cell shrinkage [180]  PS externalisation [34, 66, 93, 180]  Maintenance of plasma membrane integrity [66, 148, 180]  Decrease in ΔΨm [45, 66]  DNA fragmentation [22, 45, 66, 148, 180]  DNA ladder formation [148]  Chromatin marginalisation [148]  Chromatin condensation [66]  Entry of Ca^2+^ to the nucleus [22]  Elevation of ROS [93]  Elevation of intracellular Ca^2+^[93]  Cell-cycle deregulation [147]  *De novo* gene expression [149]  Prohibitin and RACK upregulation [150]  Induction of the SLS pathway [93] |
| ***T.cruzi*** | **Drugs:**  Aromatic diamidines [27]  Geneticin [160]  Cinnamic acid derivative [43]  Cyclopalladate complex (7a) [35]  **Other triggers:**  Antibodies [72]  Fresh serum [19, 21, 73]  Heat shock [73]  Low cell culture [73]  L-aminoacid oxidase activity [16] in bothrops jararaca venum [134]  Nutrient deprivation [68]  Stationary phase of growth [68, 160] | Cell shrinkage [16, 68, 134]  PS externalisation [27, 35, 68, 72]  Maintenance of plasma membrane integrity [35]  Decrease in ΔΨm [19, 21, 43, 134]  Mitochondrial swelling [16, 35, 134]  Mitochondrial Ca^2+^ overload [19]  Mitochondrial superoxide production [21]  Inhibition of respiration [21]  Caspase-like activity [68, 72, 134]  DNA fragmentation [16, 27, 35, 68, 72, 73, 134]  DNA ladder formation [35]  Chromatin condensation [27, 72]  ROS elevation [19, 21, 43]  Decrease in glutathione levels [21]  Decrease in thiol equivalents[21]  Nuclear translocation of TcEF-1 alpha [160] |
